# Antioxidant and Mineral Composition of Three Wild Leafy Species: A Comparison Between Microgreens and Baby Greens

**DOI:** 10.3390/foods8100487

**Published:** 2019-10-12

**Authors:** Anna Lenzi, Alessandro Orlandini, Roberta Bulgari, Antonio Ferrante, Piero Bruschi

**Affiliations:** 1 Department of Agriculture, Food, Environment and Forestry, University of Florence, 50144 Florence, Italy; piero.bruschi@unifi.it; 2 CREA Research Centre for Vegetable and Ornamental Crops, Council for Agricultural Research and Economics, 51017 Pescia, Italy; alessandro.orlandini.ao@gmail.com; 3 Department of Agricultural and Environmental Sciences–Production, Landscape, Agroenergy, University of Milan, 20133 Milano, Italy; roberta.bulgari@unimi.it (R.B.); antonio.ferrante@unimi.it (A.F.)

**Keywords:** wild plants, vegetable specialty products, immature greens, nitrate, minerals, dietary value, health risk

## Abstract

Wild plants may play an important role in human nutrition and health and, among them, many are the leafy species. We hypothesized that the wild greens could be profitably grown as microgreens and baby greens, specialty products whose market is increasing. We compared three wild leafy species (*Sanguisorba minor* Scop., *Sinapis arvensis* L., and *Taraxacum officinale* Weber ex F. H. Wigg.) harvested at the microgreen and baby green stages. Seedlings were grown hydroponically in a half-strength Hoagland nutrient solution under controlled climatic conditions. At harvest, the yield was assessed, and chlorophylls, carotenoids, anthocyanins, phenolic index, nitrate, and mineral elements were measured in the two types of product. The potential contribution to human mineral intake was calculated, and the possible risk due to the presence of metals potentially detrimental for health was estimated. Results showed that micro/baby greens of the studied wild plants achieved competitive yields and could contribute to the dietary intake of macroelements, microelements, and non-nutrient bioactive compounds. On the other hand, the wild greens showed high amounts of nitrate and traces of some metals potentially detrimental for health, suggesting the need for caution in the use of wild species for producing microgreens and baby leaves.

## 1. Introduction

Wild foods include leaves, flowers, fruits, and seeds gathered from spontaneous plants. In Europe, their consumption, often considered as an emergency practice associated with food shortage periods, has been almost completely neglected in the last decades. Nowadays wild food plants are gaining renewed attention for their nutritional value and their use is promoted by health-oriented people in the healthy lifestyle framework, with special reference to wild-green centered cuisines [1]. The leafy plants, also known as wild greens, have been traditionally consumed as salad, soup or vegetable dishes and have represented an important part of the daily diet in the Mediterranean countries, especially during the early spring and in the autumn [1]. Wild greens are known to be a good source of protein and fat, vitamins, sugars, and minerals [2,3,4]. A wide variety of phytochemicals with antioxidant effects have been also reported in many of these species [5]. Moreover, some studies demonstrated that wild plants often contain molecules showing antimicrobial potential [6] and other biological-pharmacological activities [7]. For this reason, some wild greens have recently attracted considerable attention as a source of functional foods or fortified food additive powders. On the other hand, most of them grow in anthropogenically disturbed sites such as farmlands (weeds), places of human habitation (ruderals), borders of paths and roads, etc., in soils often rich in nitrate or contaminated by metallic trace elements [8] whose detrimental effects on human health are known [9,10,11]. Considering that, due to the efficiency in root-to-shoot translocation paths, the synanthropic plants can accumulate high levels of pollutants in the aerial parts [12,13], their use as food may also entail health risks.

More than 600 wild species are used in traditional rural Italian cuisine and, among them, approximately 200 are the leafy plants [14]. We hypothesized that these wild greens could be profitably grown as specialty crops like microgreens or baby greens, with the dual advantage of widening the range of these products and, at the same time, promoting the wild species.

Microgreens are tender immature greens harvested within 10–20 days from seedling emergence and about 5 cm in height, when cotyledons are fully expanded, and the first pair of true leaves are more or less developed. Recently, microgreens have been gaining more and more popularity as a novel culinary ingredient used to enhance salads and other dishes in color, taste or texture [15], and their price may exceed $100 per kg [16]. Also, baby greens (otherwise known as baby leaves) are harvested and consumed in immature plant size, but they are older and larger than microgreens (about 10 cm in height) [16]. Baby greens are widely requested as a base component of mixed salads, especially for the ready-to-eat ones, whose consumption is constantly growing [17]. Considering both fruits and vegetables, the market for fresh-cut products in Europe has shown a double-digit growth since they began to be commercialized in the early 1980s [18]. In the United States, ready-to-eat salad mixes went through a five-fold increase in supermarket sales over a period of 20 years [19].

As reviewed by different authors [20,21,22], several studies have recently shown that plants at the microgreen stage are particularly rich in antioxidants and other health-promoting compounds, which is a reason why microgreens have started to be appreciated also as functional food. However, literature on the chemical composition of microgreens [23,24,25,26,27], as well as of baby greens [17,28], is by far focused on cultivated species, while very few studies have been carried out on wild edible plants [29,30,31]. Furthermore, the concentration of minerals and organic bioactive compounds of micro/baby greens has often been compared with that of the mature counterparts [30,32,33,34,35], while to our knowledge only one study is available about the differences in the mineral composition between microgreens and baby greens of the same species [36].

Based on this background, the aim of the present study was to evaluate three wild leafy species (*Sanguisorba minor* Scop., *Sinapis arvensis* L., and *Taraxacum officinale* Weber ex F. H. Wigg.) as possible candidates as microgreens and baby greens. Plants were grown hydroponically until they reached the microgreen or baby leaf stage, and yield, some antioxidants, nitrate, and mineral content were analyzed. The possible contribution of the different products to human mineral requirements was calculated and the health risk due to the ingestion of heavy metals possibly resulting from their consumption was also estimated.

## 2. Materials and Methods

### 2.1. Plant Material and Growth Conditions

Seeds of *S. minor* (small burnet), *S. arvensis* (wild mustard), and *T. officinale* (common dandelion) were used as starting material. *S. minor* and *S. arvensis* seeds were provided by “B & T World Seeds” (Aigues-vives, France), while seeds of *T. officinale* were harvested in late April from wild plants growing in uncultivated land in the peri-urban area of Lucca (Tuscany Region, Italy). Prior to use, seeds were surfaced-sterilized in 2.2% hypochlorite for 15 min and then rinsed under tap water for 2 min. Besides this, 1000-seed weight and germination percentage were determined (Table 1). Seeds were sown in polystyrene cell trays (27.0 × 53.5 cm^2^, 392 cells) filled with vermiculite (Asfaltex S.A., Sant Cugat del Vallés, Barcelona, Spain). Seed amount was calculated based on 1000-seed weight and germination percentage in order to obtain about eight plants per cell. After sowing, trays were kept in the dark at 20 °C for 48 h and then moved in a growth chamber at 25 ± 2 °C (day) and 17 ± 2 °C (night) with a photoperiod of 16 h under fluorescent lighting units OSRAM L36W/77 (36 WATT, 120 cm in length, 26 mm in diameter, four per tray). Trays were placed in polyethylene tanks containing 5 L of half-strength Hoagland’s nutrient solution prepared with distilled water (macroelements expressed in mM and microelements in μM: N 7.5, P 0.5, K 3.0, Ca 2.5, Mg 1.0, Fe 25.0, B 23.1, Mn 4.6, Zn 0.39, Cu 0.16, Mo 0.06; pH: 5.56; CE: 1.12 mS/cm) and arranged in a randomized block design with three replicates (1 replicate = 1 tank). The volume of the nutrient solution consumed by the crops was reintegrated at least once a week.

### 2.2. Harvesting and Yield Assessment

At the microgreen stage (first true leaf, green and swollen cotyledons), which was reached 14 days after sowing in *S. arvensis* and 16 days after sowing in both *S. minor* and *T. officinale*, half of the plants were harvested by cutting them with scissors just above the surface of the growing medium. The remaining plants were thinned to one plant per cell and leaves were harvested by cutting them with scissors after plants had reached the baby leaf stage (5–6 true leaves), 35 days after sowing in *S. arvensis* and *T. officinale*, and 43 days after sowing in *S. minor*. Microgreens and baby greens were weighed to determine yield, which was expressed in kg FW/m^2^.

### 2.3. Analysis

Harvested microgreens and baby greens were analyzed for the following chemical parameters: chlorophylls, carotenoids, phenols, anthocyanins, nitrate and mineral composition (Ca, Mg, P, Fe, Cu, Zn, Mn, Cr, Se, Mo, Co, Al, Ni, As, Cd, Pb).

#### 2.3.1. Total Chlorophyll and Carotenoids

Chlorophylls and carotenoids were extracted from fresh tissues (about 200 mg) using methanol 99.9% as solvent. Samples were kept in a dark room at 4 °C for 24 h. Quantitative chlorophyll determinations were carried out immediately after extraction. Absorbance readings were measured at 665.2 and 652.4 nm for chlorophyll *a* (Chl *a*) and *b* (Chl *b*), respectively, and 470 nm for total carotenoids. Chlorophyll and carotenoid concentrations were calculated by Lichtenthaler’s formula [37].

#### 2.3.2. Phenolic Index and Anthocyanins Concentration

Samples of frozen tissue (30–50 mg) were ground in pre-chilled mortar and extracted into methanolic HCl (1%). After that, they were incubated overnight at 4 °C, in the dark. Phenols were spectrophotometrically determined by measuring directly the methanolic extract absorbance at 320 nm (phenolic index), slightly modifying the procedures reported in Ferrante et al. [38]. The phenolic index was expressed as ABS_320nm_/g FW [38]. For anthocyanins determination, the concentration of cyanidin-3-glucoside equivalents was determined spectrophotometrically at 535 nm [39]. The same methanolic extract was used for both determinations.

#### 2.3.3. Nitrate

Nitrate content was measured with the salicylsulphuric acid method [40]. 10 mg of oven-dried samples (80 °C for 48 h) were suspended in 10 mL of distilled water and left in agitation for 2 h. After that, 20 μL of sample were added to 80 μL of 5% salicylic acid in sulphuric acid and to 3 mL of NaOH 1.5 N. Samples were cooled at room temperature and the spectrophotometer readings were performed at 410 nm. Nitrate content was calculated referring to a KNO_3_ standard calibration curve. Data were expressed on a fresh weight (FW) basis considering the fresh weight/dry weight ratio.

#### 2.3.4. Mineral Composition

For assessing the mineral composition, oven-dried samples (80 °C for 48 h) were ground and digested with nitric acid, and elements were measured using inductively coupled plasma mass spectroscopy (ICP-MS). Data were expressed on an FW basis considering the fresh weight/dry weight ratio.

### 2.4. Contribution to Mineral Dietary Intake and Health Risk Assessment

The estimated dietary intake (EDI, mg/day) of mineral elements possibly resulting from the consumption of micro/baby greens of the studied species was calculated by the following formula:

EDI = C_metal_ × (SP/1000) (1)

where,

C_metal_ = the element concentration (mg/kg FW) in the produce (2)

SP = a supposed portion of 20 g of micro/baby greens
(3)

For evaluating the contribution of microgreens and baby greens to human mineral requirements, EDI was expressed as percentage (EDI%) of the recommended dietary intake (RDI, mg/day) (for Ca, P, Mg, Fe, Cu, Zn, Mo, and Se) or adequate intake (AI, mg/day) (for Mn and Cr) as defined by Italian Society of Human Nutrition (SINU), considering RDI and AI values referred to an adult male [41].

In order to assess the possible health risk due to the intake of metals related to micro/baby greens consumption, the health risk index (HRI) was calculated for Fe, Cu, Zn, Mn, Cr, Se, Mo, Co, Ni, As, and Cd according to the following formula:

HRI = EDI_Bw_/RfD (4)

where,

EDI_Bw_ = EDI (as defined above) per kg of body weight (BW) (5)

RfD (mg/kg BW/day) = oral reference dose
(6)

which is an estimate of the daily exposure of humans to heavy metals having no hazardous effect during the lifetime according to US-EPA [42]

As BW an average body weight for an adult was considered and assumed to be 55.9 kg as in previous studies [43]. Since RfD is not available for Al and Pb, the possible health risk was evaluated on the basis of Al tolerable weekly intake (TWI; mg/kg BW/week) according to EFSA [44], and of Pb *Codex Alimentarius* maximum level (ML; mg/kg FW) (maximum concentration of a contaminant in a food commodity recommended by the FAO/WHO *Codex Alimentarius* Commission to be legally permitted in that commodity) [45].

### 2.5. Statistical Analysis

Yield and composition data were subjected to a two-way ANOVA (3 species × 2 stages of harvest) according to a randomized block experimental design with three replicates, by using CoStat Statistics Software. Significant differences among means were determined by using Duncan’s Test at *p* < 0.05. A principal component analysis (PCA) was also performed on composition data by using the software STATISTICA for Windows. Before performing PCA, all values of considered variables were replaced by standardized values, which were computed as follows:

Standardized value = (raw value − mean)/Std. deviation
(7)

## 3. Results

### 3.1. Yield

**Figure 1:**
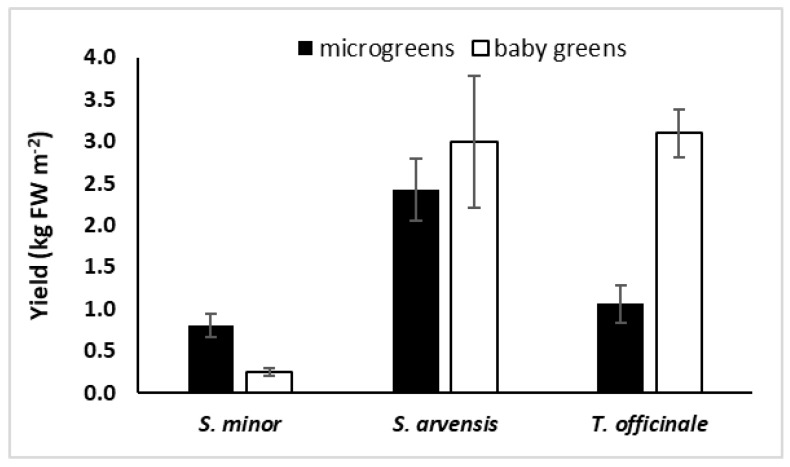
Yield of *S. minor*, *S. arvensis*, and *T. officinale* microgreens and baby greens grown in a hydroponic system. Data are means ± SD (*n* = 3).

Considering the average of the two stages of harvest, the most and the least productive species were *S. arvensis* (2.41 kg FW/m^2^) and *S. minor* (0.39 kg FW/m^2^), respectively. An intermediate yield was obtained in *T. officinale* (1.83 kg FW/m^2^). On average, the three species resulted in higher yield when they were harvested at the baby leaf stage (2.11 kg FW/m^2^) rather than as microgreens (0.99 kg FW/m^2^). A significant interaction species × stage of the harvest was observed (*F* = 24.66; *p* < 0.001), revealing that *S. minor* gave higher yield as microgreens than as baby greens, while the contrary occurred in *T. officinale* (Figure 1). In *S. arvensis*, harvesting at different stages resulted in comparable yields.

### 3.2. Chlorophylls, Carotenoids, Phenols, Anthocyanins and Nitrate Content

**Figure 2:**
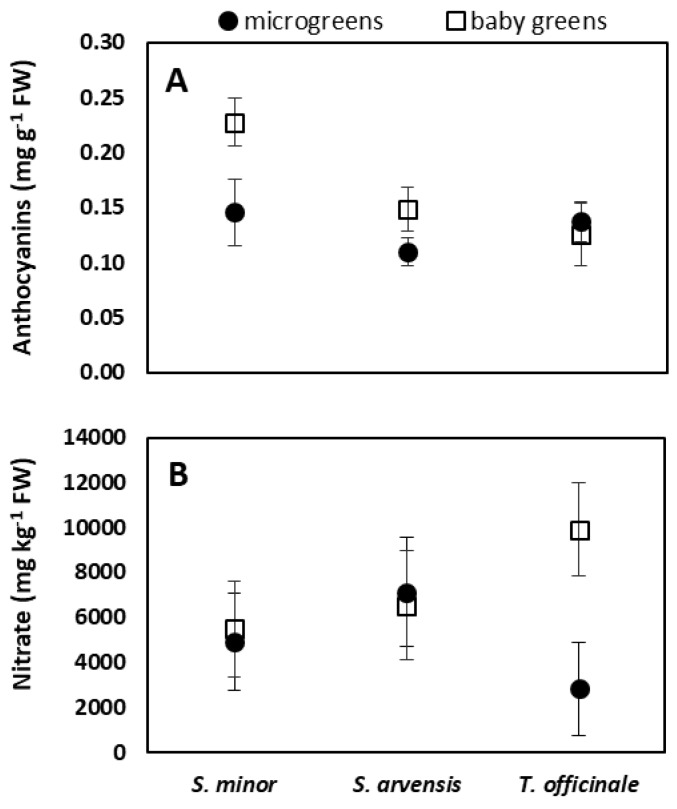
Interaction species × stage of harvest for anthocyanins (**A**) and nitrate concentration (**B**) of *S. minor*, *S. arvensis*, and *T. officinale* grown in a hydroponic system and harvested at microgreen or baby green stage. Data are means ± SD (*n* = 3).

Statistical analysis showed that chlorophylls concentration, considering both the total amount and the single chlorophyll types (Chl *a* and Chl *b*), as well as the phenol values (expressed as phenolic index), were not significantly different among the species and the stages of harvest (Table 2). For carotenoids, higher concentration was found in baby greens than in microgreens, while no differences were observed among the species (Table 2). On the contrary, the species, as well as the stages of the harvest, showed significant differences in anthocyanin concentration. Among the species, the highest anthocyanin amount was found in *S. minor* (0.19 mg/g FW); between microgreens and baby greens, the latter showed higher values (Table 2). However, the significant interaction species × stage of harvest highlighted that such difference did not occur in *T. officinale* (Figure 2A). The species did not differ in nitrate concentration, whose values, on average, ranged from 5205 mg/kg FW (*S. minor*) to 6833 mg/kg FW (*S. arvensis*) (Table 2). The comparison between the stages of harvest revealed a significantly higher nitrate concentration in baby greens than in microgreens. A significant interaction species × stage of harvest was found for nitrate content. Specifically, *T. officinale* microgreens showed much lower nitrate values than baby greens, while for *S. minor* and *S. arvensis* nitrate concentration was similar in the two product types (Figure 2B).

### 3.3. Mineral Content

**Figure 3:**
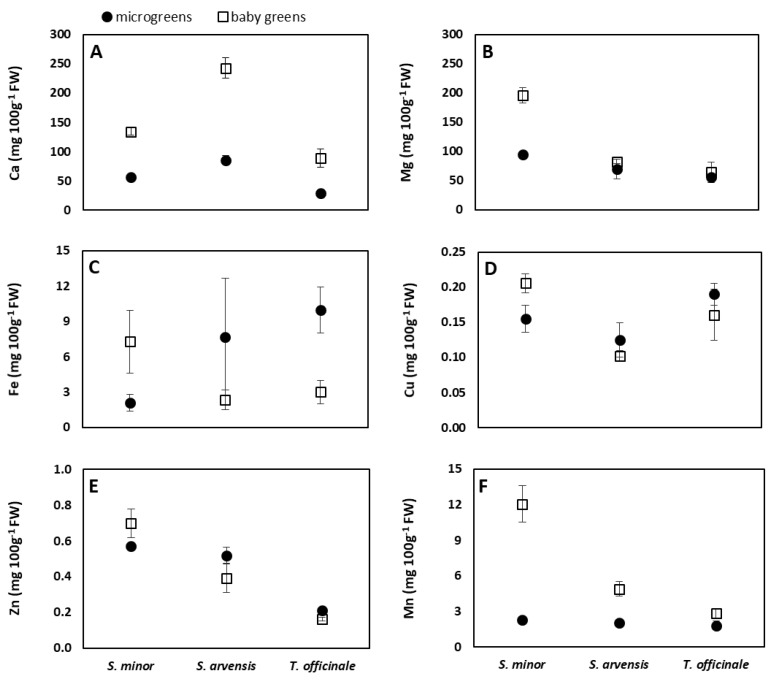
Interaction species × stage of harvest for Ca (**A**), Mg (**B**), Fe (**C**), Cu (**D**), Zn (**E**), and Mn (**F**) concentration in *S. minor*, *S. arvensis*, and *T. officinale* grown in a hydroponic system and harvested at microgreen or baby green stage. Data are means ± SD (*n* = 3).

**Figure 4:**
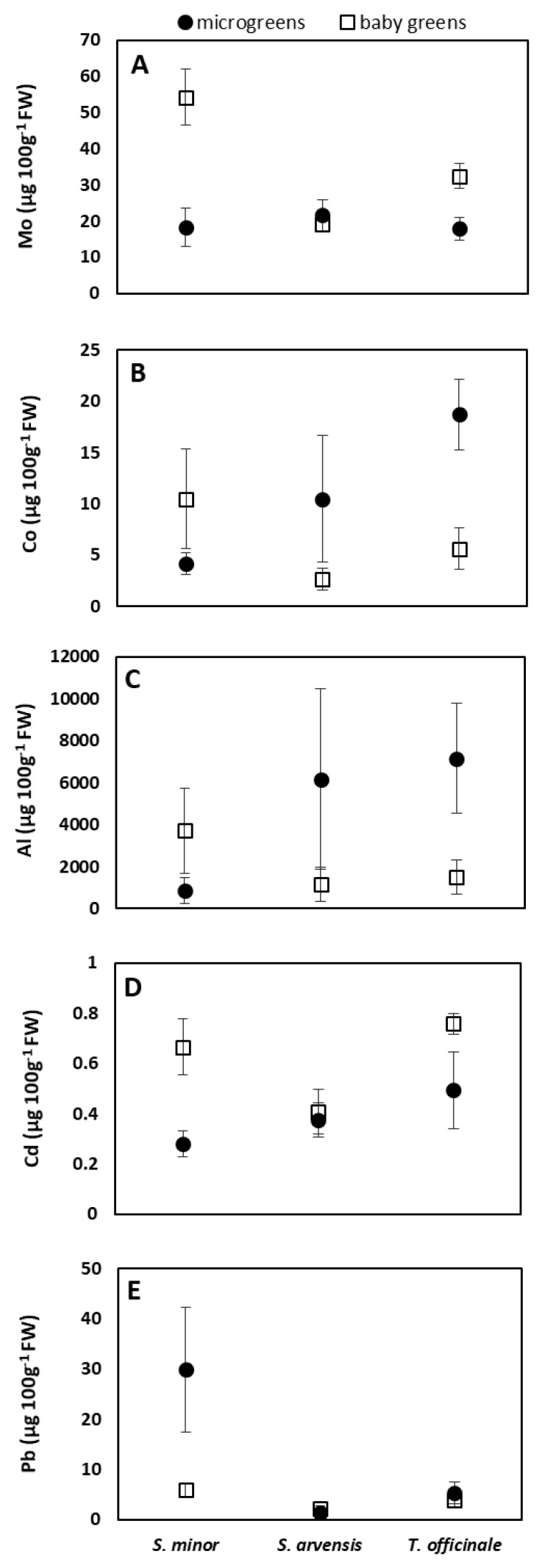
Interaction species × stage of harvest for Mo (**A**), Co (**B**), Al (**C**), Cd (**D**), and Pb (**E**) concentration in *S. minor*, *S. arvensis*, and *T. officinale* grown in a hydroponic system and harvested at microgreen or baby green stage. Data are means ± SD (*n* = 3).

Significant differences in element concentration between the species were observed for Ca, Mg, P, Cu, Zn, Mn, Se, Mo, Cd, and Pb (Table 3 and Table 4). *S. minor* was richer in Mg, P, Zn, Mn, Mo, and Pb than *S. arvensis* and *T. officinale*. The latter ones did not differ for these elements with the exception of Zn and Mn, which were higher in *S. arvensis* than in *T. officinale*. *S. arvensis* showed the highest concentration in Ca, but the lowest amount in Cu and Se, and *T. officinale* was richer in Cd. No significant differences between the species were noticed for the content in Fe, Cr, Co, Al, Ni, and As. As the average of the three species, baby greens were found to contain higher amounts of Ca, Mg, P, Mn, Mo, and Cd than microgreens, which, conversely, showed higher concentrations in Co, Al, and Pb (Table 3 and Table 4). The interaction between species and stage of the harvest was significant for Ca, Mg, Fe, Cu, Zn, Mn, Mo, Co, Al, Cd, and Pb (Table 3 and Table 4). *S. arvensis* was particularly reached in Ca and *S. minor* in Mg, Zn, Mn and Mo at the baby green stage (Figure 3A,B,E,F and Figure 4A) On the contrary, the high accumulation of Pb in *S. minor* occurred only in microgreens (Figure 4E). *S. minor* showed a higher concentration of Fe, Cu, Co and Al when harvested at the baby greens stage, while for *S. arvensis* and *T. officinale* microgreens were richer in these elements than baby greens (Figure 3C,D and Figure 4B,C). For Cd, the difference between microgreens and baby greens was observed in *S. minor* and *T. officinale* (Figure 4D).

### 3.4. Principal Component Analysis (PCA)

**Figure 5:**
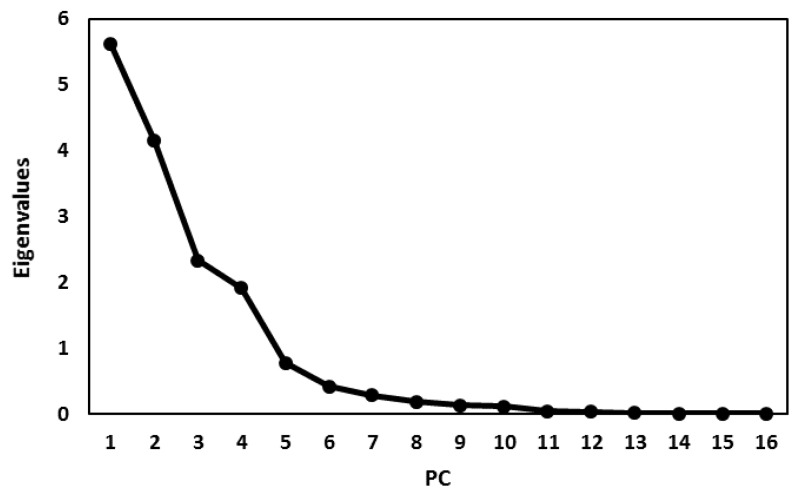
Screen plot of eigenvalues in PCA analysis.

**Figure 6:**
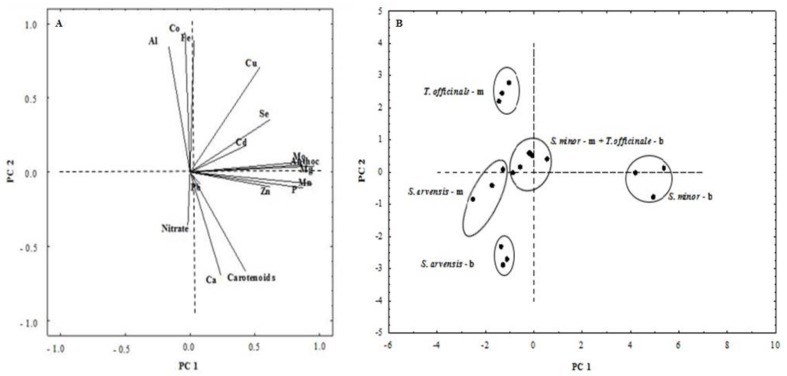
Loading plot (**A**) and scores (**B**) for each component (PC 1 and PC 2). Anthoc = anthocyanins; m and b correspond to microgreen and baby green stages, respectively.

A PCA was carried out in order to investigate whether there were factors grouping correlated variables together and to identify clusters across species and stages of harvest. Two principal components (PCs) explaining a cumulative variance of 61.0% were identified based on a screen plot of eigenvalues (Figure 5). PC 1, which explained 35.1% of the total variance, was positively correlated with anthocyanins, Mg, Mn, Mo, and P, while PC 2 (25.9% of the total variance) was negatively correlated to carotenoids and Ca and positively to Fe, Cu, Co and Al. The loading plot reported in Figure 6A illustrates the relationships between the parameters considered in this study. Parameters located close to each other had a strong co-variance. Moreover, parameters far from the origin contributed more to the PCs than parameters close to it. In the rightmost part of Figure 6A, two clusters (the first with anthocyanins, Mo and Mg, and the second with P, Mn and Zn) suggested a strong co-variance between these variables, as well as a strong contribution to PC 1. The most important variables contributing to PC 2 were Ca and carotenoids and, on the opposite side, Al, Co, Fe and Cu. The relationship existing between the analyzed samples are shown in the score plot (Figure 6B). PC 1 and PC 2 discriminated species and stages of harvest in five groups. *S. minor* baby greens were positioned in the right half of the plot (the positive side of PC 1): they were characterized by the highest levels of anthocyanins, Mg, Mn, Mo, P, and Zn. *T. officinale* microgreens were included in the upper left quadrant (the positive side of PC 2): they were characterized by high Fe, Co and Al concentrations and low nitrate content. *S. arvensis* samples harvested at the baby leaf stage were included in the lower-left quadrant (the negative side of PC 2): they were characterized by high carotenoids and Ca content. Differently, *S. arvensis* microgreens were characterized by low anthocyanins and relatively high nitrate and Al contents. Finally, *S. minor* microgreens and *T. officinale* baby greens were closely clustered at the center of the scatterplot (Figure 6B).

### 3.5. Contribution to Mineral Dietary Intake and Health Risk Assessment

The potential contribution of the analyzed microgreens and baby greens to human mineral requirements was very different for the different elements (Table 5). With reference to a portion of 20 g of microgreens/baby greens, the EDI% ranged from very low values, even lower than 1% (Ca from *T. officinale* microgreens and Zn from *S. arvensis* and *T. officinale* regardless of the stage of harvest) to values higher than 100% in the case of Cr (*T. officinale* microgreens and *S. minor* baby greens), revealing a potential intake so far over the AI of this element. Values of EDI% over 10% were detected for Mg (*S. minor* baby greens), Fe (*S. arvensis* and *T. officinale* microgreens, *S. minor* baby greens), Mn (all the species, both the stages), Se (*S. minor* baby greens) and Mo (*S. minor* and *T. officinale* baby greens). Considering the average of the three species, the EDI% values from microgreens showed the following ascending order for the different elements: Zn (0.79%), Ca (1.14%), P (1.82%), Cu (3.48%), Mg (6.11%), Se (6.58%), Mo (8.56%), Fe (13.18%), Mn (15.16%) and Cr (109.90%). Similarly, the order of EDI% from baby greens was Zn (0.76%), P (2.45%), Ca (3.10%), Cu (3.46%), Se (7.40%), Fe (8.44%), Mg (9.52%), Mo (15.67%), Mn (48.95%) and Cr (112.21%).

Regarding the assessment of the health risk related to detrimental metals present in the micro/baby greens, all the EDI_BW_ values (Fe, Cu, Zn, Mn, Cr, Se, Mo, Co, Ni, As, Cd), calculated with reference to a portion of 20 g, were smaller than the corresponding RFDs (US-EPA IRIS, 2013), and the HRIs were far below 1 (Table 6). For Al, for any species and stage of the harvest, weekly consumption of 20 g of product per day would bring to an element intake far below the TWI (1 mg/kg body weight/week) recommended by EFSA (2008) (data not shown). For Pb, the ML recommended by the FAO/WHO *Codex Alimentarius* Commission to be legally permitted in leafy vegetables (30 μg/100 g) was exceeded in *S. minor* microgreens (Figure 4E).

## 4. Discussion

The fresh biomass of *S. minor*, *S. arvensis*, and *T. officinale* microgreens (Figure 1) ranged from 0.8 kg/m^2^ (*S. minor*) to 2.4 kg/m^2^ (*S. arvensis*) and was consistent with that reported by Bulgari et al. [46], Paradiso et al. [47], and Renna et al. [48] for microgreens of vegetable crop species. Kyriacou et al. [27] found that the microgreens of 10 different species produced over 3 kg FW/m^2^, but these authors adopted a longer growth period, harvesting the microgreens at the second leaf stage. At the baby green stage, *S. arvensis* and *T. officinale* yield (about 3 kg FW/m^2^) was higher than that of cultivated species [28,49,50]. The fresh biomass of *S. minor* baby leaves was only 0.2 kg FW/m^2^. In this case, the increase in plant fresh weight from microgreens to baby leaves did not compensate for the lower plant density, suggesting that a later stage of harvest (i.e., more than 5–6 leaves) would have been more proper for *S. minor*.

Wild edible plants contain important amounts of non-nutrient compounds beneficial for health, such as carotenoids and phenolic compounds [51]. Healthy effects of these bioactive molecules are often associated with antioxidant activity, leading to the reduction in cardiovascular disease risk factors, the decrease of the incidence of cancer, and protection against a wide range of chronic diseases [52]. Besides the health benefits, carotenoids and anthocyanins influence the organoleptic quality of plant products (taste, aroma) and their visual appearance [27,53]. Together with chlorophylls, they are the main pigments contributing to leaf color, which is particularly important for leafy vegetables since it strongly conditions the evaluation by the consumer and, especially in produce like microgreens and baby leaves, should be uniform and intense [38,54].

Considering the microgreen stage, the three studied wild species showed usually higher or, sometimes, comparable chlorophyll, carotenoids and anthocyanin concentrations than those of most vegetable crop species analyzed in previous studies [25,27,46,47,55,56]. Nevertheless, under LED illumination some microgreens of Brassicaceae family showed even higher carotenoid amounts [57], and particularly high contents of total anthocyanins were measured by Samuolienė et al. [26] in the microgreens of 10 vegetable species. As reviewed by Saini et al. [17] and Di Gioia et al. [58], many studies have shown that baby greens are a good source of antioxidants. To our knowledge, no comparison between baby greens and microgreens of the same species has been carried out on this aspect yet. Among the species we analyzed, *S. minor* showed the highest anthocyanin amounts, and baby greens were richer in these compounds, as well as in carotenoids than microgreens (Table 2 and Figure 2). Considering that, in general, these phytochemicals increase during leaf development and reach the maximum level in mature leaves [59] this result is probably ascribable to the different stage of the harvest of the two products. For the same reason, the lower content of carotenoids found in *S. minor* and *T. officinale* micro/baby greens in comparison with values reported in the literature for adult plants of these species [51] is reasonable.

Microgreens and baby greens of vegetable crops show very variable nitrate contents [27,28]. Such variability is due to the different accumulation ability of the different genotypes, but it is also strongly influenced by agronomic and environmental factors [9]. When microgreens were compared to adult plants of the same species grown in the same conditions, lower nitrate content was observed in microgreens [34]. Accordingly, in our study, the more mature stage (baby greens) of *T. officinale* contained more nitrate than the microgreen counterpart. Conversely, compared to nitrate content measured in *T. officinale* adult leaves collected in the wild [60], we found much higher values, probably due to higher nitrogen availability in the nutrient solution than in the uncultivated soil. In *S. minor* and *S. arvensis*, no differences were found between microgreens and baby leaves (Figure 2).

Concerns about nitrate accumulation in vegetables are mainly related to the fact that nitrate ingestion is thought to be a risk factor for stomach cancer [9]. That has brought the EU Commission to establish maximum nitrate levels allowed for the commercialization of some vegetables (spinach, lettuce, and rocket) ranging from 2000 to 7000 mg/kg FW (Regulation No 1258/2011). On the other hand, the association between the estimated intake of nitrate in the diet and stomach cancer has been recently rejected on the basis of the review of the epidemiological literature [10]. Moreover, different authors have reported that a diet high in nitrate is beneficial to humans for cardiovascular and cerebrovascular health [61,62], in particular in older adults [63]. In our study, nitrate concentration was over 2000 mg/kg FW in all the analyzed samples, and in *S. arvensis* microgreens and *T. officinale* baby greens exceed 7000 mg/kg FW. If, on one hand, that can be considered a limitation for these products, on the other hand, it makes them possible candidates to provide dietary nitrate supplementation for some categories of people like the elderly.

Data available in the literature demonstrate that wild edible plants may be an excellent source of macro and microelements for humans. Wild greens usually contribute to the dietary intake of minerals more than wild fruits, and for Ca, Mg, Fe, and Mn, the provided amounts may even reach half of the recommended daily requirement [4]. In *S. arvensis*, *S. minor* and *T. officinale* micro/baby greens, analyzed in this study, these elements showed concentrations sometimes higher and sometimes lower than those reported in the literature for adult counterparts [4,64,65]. In previous studies, microgreens were found to contain lower Ca amount than adults in amaranth [30] and kale [36], while the contrary was found in lettuce [34], and broccoli grown on compost [66]. Among the three analyzed species, *S. arvensis* showed higher Ca concentrations than *S. minor* and *T. officinale*, and, at the baby green stage, exceeded 200 mg/100 g FW (Figure 3A), which is considered a good Ca content [4]. In all the three species, baby greens were richer in Ca than microgreens (Table 3 and Figure 3A), confirming the results of Waterland et al. [36] in kale. These authors found that kale baby greens contained also higher amounts of Mg and Fe than microgreens of the same species. In our study, baby greens were richer in Mg than microgreens only in *S. minor* (Figure 3B). This species, on average, showed much more Mg than *S. arvensis* and *T. officinale* (Table 3). That is not surprising, considering that among wild edible greens, *S. minor* is considered one of the richest Mg sources [4]. Furthermore, *S. minor* needed eight days more than *S. arvensis* and *T. officinale* to reach the baby leaf stage and the different growth period could have affected the mineral composition [24]. In comparison with microgreens [24,27,47] and baby greens [28,67] of many vegetable crop species, the wild greens grown in our study showed medium to low content as microgreens and medium to high content as baby greens for Ca and contained medium to high amounts of Mg at both stages of harvest. For Fe, according to what was observed by Waterland et al. [36] in kale, *S. minor* baby greens showed higher concentration than microgreens, while the opposite occurred in *T. officinale* (Figure 3C). It is interesting to notice that, considering the reviewed literature on wild greens [2,4,68], vegetable microgreens [24,30,34,46,47], and vegetable baby greens [67] of different species, *T. officinale* microgreens exceeded the Fe amount of any of them. Some differences among species were observed in P, Cu, Zn, and Mn concentrations, and for P and Mn also between stages of harvest (Table 3 and Figure 3D–F). For all the three species and both the stages, values were comparable (P and Zn) or higher (Mn and Cu) than those measured by other authors in vegetable microgreens [24,47] or baby greens [67]. Waterland at al. [36], noticed higher Zn amounts in kale baby leaves in comparison with the microgreen counterparts. Contrasting this, we did not observe differences in Zn concentration between the two stages of harvest.

According to the Regulation (EU) No. 1169/2011 on the provision of food information to consumers, foods can be considered significant sources of mineral elements if they contain, per 100 g, at least 15% of the reference values reported in the Annex XIII, and corresponding to (in mg): 120.0 (Ca), 56.3 (Mg), 105.0 (P), 2.10 (Fe), 0.15 (Cu), 1.50 (Zn), 0.30 (Mn), 0.0060 (Cr), 0.0083 (Se), and 0.0075 (Mo). The comparison between these amounts and data shown in Table 3, Table 4 and Figure 3, Figure 4 would indicate that micro/baby greens of the wild species analyzed in our study should be good sources of several minerals in the human diet. Nevertheless, for evaluating their contribution it cannot be disregarded that specialty produce, especially microgreens, are normally consumed in small amounts. Therefore, in order to avoid overestimations, in our study EDI% was calculated for a portion of 20 g (Table 5), which was considered quite a reasonable amount for the comparison between microgreens and baby greens. As reference values, RDI or AI as defined in the Materials and Methods section were considered. The largest contributions were observed for Cr, Mn, Mo, Mg, and Fe. For the latter, particularly noticeable was the EDI% of *T. officinale* microgreens (almost 20%). Intermediate EDI% values were noticed for Se and Cu, and the lowest for Zn, Ca, and P. Zinc and P data are consistent with the fact that leafy vegetables, either wild or cultivated, do not stand out by their P and Zn concentrations, and thus they are not generally recognized as good sources of these elements [4].

Minor elements (Cr, Se, Mo, Co, Al, Ni, As, Cd, and Pb) have been rarely measured in micro/baby greens. Molybdenum concentration in lettuce microgreens [34] was comparable to the values found in the microgreens of the wild species considered in our study but lower than those of *S. minor* and *T. officinale* harvested at the baby stage (Figure 4A). For Se, the wild greens, independently from the stage of harvest, showed higher amounts than those measured in lettuce microgreens [34], but *S. minor* and *T. officinale* were richer in this element than *S. arvensis* (Table 4). Xiao et al. [24] investigated Cd and Pb content of 30 vegetable microgreens of the Brassicaceae family, finding that these elements were under the limit of detection. Also, Paradiso et al. [47] observed that Pb was under the detection limit in some genotypes of microgreens belonging to Brassicaceae or Asteraceae, while in the same samples Cd concentration was about 10 times over the values observed in our study (Figure 4D). The species considered in our study resulted to contain Pb, and, in *S. minor* microgreens, the amount of this metal exceeded the ML of 30 μg/100 g FW recommended by the FAO/WHO *Codex Alimentarius* Commission for leafy vegetables [45]. Other heavy metals detected in the wild greens were Cr, Co, Al, Ni, and As. That was not surprising since ruderal species, like *S. minor*, *S. arvensis* and *T. officinale*, are well-known for their capability to accumulate contaminants, especially in leaves [8,12,13]. For example, Giacomino et al. [69] and Stark et al. [70] found potentially hazardous levels of Pb and As, respectively, in some samples of spontaneously growing *T. officinale*, and *S. arvensis* stood out among different wild species for Cd and Cr accumulation in both contaminated and not-contaminated soils [12,64]. In our study, microgreens and baby greens were grown in a controlled environment and hydroponically, using a nutrient solution prepared with distilled water, therefore it can be supposed that the detected trace elements derived from the mineral fertilizers used to prepare the nutrient solution [71,72] and from vermiculite used as growing medium [73]. However, since HRI values <1 are assumed to be safe in terms of population exposure to metals [43], HRI calculated for Fe, Cu, Mn, Cr, Se, Mo, Co, Ni, As and Cd considering a portion of 20 g, being far below 1 (Table 6), excluded health risks due to the consumption of micro/baby greens in relation to these elements. Health risks were excluded also for Al, whose ingestion was calculated on a weekly basis according to EFSA recommendation [44]. Even considering portions of 100 g, which are quite improbable for these products, HRI values would still be below 1 in most cases. Only *S. minor* baby greens and *T. officinale* microgreens would show HRI >1 for Mn and Cr, and for Co and Cr, respectively, if EDI_Bw_ was reported to 100 g product.

## 5. Conclusions

The results of this study showed that *S. minor*, *S. arvensis*, and *T. officinale* would be interesting species for producing specialty crops like microgreens and baby greens. Actually, not only did they achieve competitive yield, but also demonstrated that their contribution to the dietary intake of macroelements, microelements, and non-nutrient bioactive compounds would be comparable, or even larger, than that of vegetable crop species. Among the species, *S. minor* showed the highest amounts of Mg, P, Zn, Mn, and Mo, and *T. officinale* microgreens stood out by Fe content. Between microgreens and baby greens, the latter were often richer in minerals and antioxidants. On the other hand, the wild greens showed high amounts of nitrate, which could be a limitation for commercialization, and the presence of some metals potentially detrimental for human health. Although micro/baby greens are normally consumed in small portions, and the calculated HRI values were far below 1, such a finding suggests caution. Therefore, the aspect of the accumulation ability of wild ruderal species should always be considered prior to introducing them in cultivation, and, in this case, strict control of possible sources of chemical contamination (water, salts used to prepare the nutrient solution, and substrates) would be necessary.

## Figures and Tables

**Table 1:** One thousand-seed weight and germination rate of *Sanguisorba minor* Scop., *Sinapis arvensis* L., and *Taraxacum officinale* Weber ex F. H. Wigg. seeds.

Species	1000-Seed Weight ^1^ g	Germination ^2^ (%)
*S. minor*	7.02 ± 0.30	75.5 ± 3.4
*S. arvensis*	2.48 ± 0.13	61.0 ± 3.7
*T. officinale*	0.62 ± 0.03	72.0 ± 2.9

^1^ Means of eight samples of 100 seeds each × 10 ± SD. ^2^ Means ± SD of four samples of 50 seeds each, kept in the dark at 20 °C for 21 days.

**Table 2:** Chlorophylls (Chl *a*, Chl *b* and total), carotenoids, phenols, anthocyanins, and nitrate concentrations of *S. minor*, *S. arvensis*, and *T. officinale* grown in a hydroponic system and harvested at microgreen or baby green stage.

Treatments	Chl *a* mg/g FW	Chl *b* mg/g FW	Chl *a*+*b* mg/g FW	Carotenoids mg/g FW	Anthocyanins ^1^ mg/g FW	Phenolic Index ABS_320 nm_/g FW	Nitrate mg/kg FW
**Species**							
*S. minor*	0.84 ± 0.58	0.66 ± 0.58	1.41 ± 1.17	0.16 ± 0.07	0.19 ± 0.05 a	11.95 ± 2.85	5205 ± 2023
*S. arvensis*	1.00 ± 0.40	0.55 ± 0.35	1.55 ± 0.68	0.18 ± 0.08	0.13 ± 0.03 b	10.98 ± 2.51	6833 ± 1626
*T. officinale*	0.90 ± 0.38	0.65 ± 0.31	1.55 ± 0.64	0.11 ± 0.04	0.13 ± 0.02 b	10.78 ± 1.91	6368 ± 4100
**Stage of harvest**							
Microgreens	0.76 ± 0.38	0.50 ± 0.50	1.26 ± 0.85	0.11 ± 0.05 b	0.13 ± 0.02 b	10.30 ± 2.74	4962 ± 2231 b
Baby greens	1.07 ± 0.46	0.74 ± 0.26	1.75 ± 0.74	0.20 ± 0.05 a	0.17 ± 0.05 a	12.17 ± 1.54	7308 ± 2774 a
**Significance**							
Species	ns	ns	ns	ns	***	ns	ns
Stage of harvest	ns	ns	ns	**	**	ns	*
Species x stage of harvest	ns	ns	ns	ns	*	ns	*

^1^ Cyanidin-3-glucoside equivalent. Means (± SD) in columns not sharing the same letters are significantly different according to Duncan’s Test (*p* ≤ 0.05). ns = not significant; asterisk(s) = significant at 0.05 (*), 0.005 (**) or 0.001(***) level of significance.

**Table 3:** Calcium, Mg, P, Fe, Cu, Zn, and Mn concentration in *S. minor*, *S. arvensis*, and *T. officinale* grown in a hydroponic system and harvested at microgreen or baby green stage.

Treatments	Ca	Mg	P	Fe	Cu	Zn	Mn
	mg/100 g FW						
**Species**							
*S. minor*	95.59 ± 42.59 b	145.58 ± 55.71 a	108.11 ± 13.76 a	4.70 ± 3.32	0.18 ± 0.03 a	0.63 ± 0.09 a	7.16 ± 5.45 a
*S. arvensis*	163.95 ± 87.09 a	75.99 ± 13.21 b	54.08 ± 11.56 b	5.03 ± 4.31	0.11 ± 0.02 b	0.46 ± 0.09 b	3.48 ± 1.61 b
*T. officinale*	59.00 ± 34.22 c	59.79 ± 12.78 b	61.56 ± 20.07 b	6.48 ± 4.04	0.18 ± 0.03 a	0.19 ± 0.03 c	2.34 ± 0.67 c
**Stage of harvest**							
Microgreens	57.05 ± 24.60 b	73.39 ± 19.71 b	63.59 ± 25.16 b	6.59 ± 4.40	0.16 ± 0.03	0.43 ± 0.17	2.05 ± 0.30 b
Baby greens	155.31 ± 69.48 a	114.18 ± 62.79 a	85.57 ± 28.89 a	4.22 ± 2.74	0.16 ± 0.05	0.42 ± 0.24	6.61 ± 4.27 a
**Significance**							
Species	***	***	***	ns	***	***	***
Stage of harvest	***	***	**	ns	ns	ns	***
Species x stage of harvest	***	***	ns	**	**	**	***

Means (± SD) in columns not sharing the same letters are significantly different according to Duncan’s Test (*p* ≤ 0.05). ns = not significant; asterisk(s) = significant at 0.05 (*). 0.005 (**) or 0.001(***) level of significance.

**Table 4:** Chromium, Se, Mo, Co, Al, Ni, As, Cd, and Pb concentration in *S. minor*, *S. arvensis*, and *T. officinale* grown in a hydroponic system and harvested at microgreen or baby green stage.

Treatments	Cr	Se	Mo	Co	Al	Ni	As	Cd	Pb
	μg/100 g FW								
**Species**									
*S. minor*	256.4 ± 281.7	25.3 ± 4.2 a	36.3 ± 20.5 a	7.3 ± 4.7	2284.8 ± 2058.7	138.5 ± 130.2	2.4 ± 0.7	0.5 ± 0.2 b	17.9 ± 15.3 a
*S. arvensis*	59.3 ±5 1.1	10.0 ± 3.2 b	20.3 ± 3.2 b	6.6 ± 5.8	3664.8 ± 3903.1	32.3 ± 28.1	1.0 ± 0.3	0.4 ± 0.1 b	1.8 ± 0.6 b
*T. officinale*	267.3 ± 275.9	22.3 ± 4.1 a	25.2 ± 8.5	12.2 ± 7.6	4331.3 ± 3546.0	139.8 ± 146.8	4.1 ± 5.7	0.6 ± 0.2 a	4.6 ± 1.9 b
**Stage of harvest**									
Microgreens	192.3 ± 243.0	18.1 ± 5.8	19.3 ± 4.2 b	11.1 ± 7.3 a	4729.6 ± 3878.4 a	107.9 ± 125.2	1.3 ± 0.5	0.4 ± 0.1 b	12.2 ± 14.8 a
Baby greens	196.4 ± 245.6	20.4 ± 9.5	35.2 ± 16.0 a	6.3 ± 4.3 b	2124.3 ± 1674.4 b	99.2 ± 120.5	3.6 ± 4.6	0.6 ± 0.2 a	4.0 ± 1.9 b
**Significance**									
Species	ns	***	***	ns	ns	ns	ns	**	***
Stage of harvest	ns	ns	***	*	*	ns	ns	***	**
Species x stage of harvest	ns	ns	***	**	*	ns	ns	*	**

Means (± SD) in columns not sharing the same letters are significantly different according to Duncan’s Test (*p* ≤ 0.05). ns = not significant; asterisk(s) = significant at 0.05 (*). 0.005 (**) or 0.001(***) level of significance.

**Table 5:** Estimated dietary intake expressed as percentage (EDI%) of the recommended dietary intake (RDI) or adequate intake (AI) resulting from the consumption (20 g per day) of microgreens or baby greens of *S. minor*, *S. arvensis*, and *T. officinale*.

Mineral	RDI/*AI* ^1^ mg/day	Microgreens			Baby Greens		
		*S. minor*	*S. arvensis*	*T. officinale*	*S. minor*	*S. arvensis*	*T. officinale*
Ca	**1000**	1.14	1.7	0.58	2.69	4.85	1.78
Mg	**240**	7.94	5.79	4.61	16.32	6.88	5.35
P	**700**	2.76	1.28	1.41	3.42	1.81	2.11
Fe	**10**	4.23	15.39	19.91	14.56	4.73	6.02
Cu	**0.9**	3.44	2.78	4.22	4.56	2.26	3.57
Zn	**11**	1.04	0.95	0.39	1.27	0.71	0.30
Mn	*2.7*	16.82	15.28	13.39	89.32	36.30	21.23
Cr	*0.035*	42.27	54.46	232.98	250.78	13.33	72.51
Se	**0.055**	8.16	4.24	7.35	10.26	3.05	8.88
Mo	**0.045**	8.15	9.60	7.94	24.09	8.48	14.43

^1^ RDI (bold) and AI (italic) according to SINU (2014).

**Table 6:** Estimated daily intake per kg of body weight (EDI_BW_, mg/kg body weight/day) and health risk index (HRI) resulting from the consumption (20 g per day) of microgreens or baby greens of *S. minor*, *S. arvensis*, and *T. officinale*.

Metal		Microgreens			Baby Greens		
		*S. minor*	*S. arvensis*	*T. officinale*	*S. minor*	*S. arvensis*	*T. officinale*
Fe (R*f*D = 0.7)	**EDI_BW_**	0.007565	0.027538	0.035613	0.026045	0.008454	0.010773
	**HRI**	0.010808	0.039339	0.050876	0.037207	0.012077	0.015391
Cu (R*f*D = 0.01)	**EDI_BW_**	0.000553	0.000447	0.00068	0.000735	0.000363	0.000575
	**HRI**	0.013835	0.011178	0.016994	0.018383	0.009082	0.014373
Zn (R*f*D = 0.3)	**EDI_BW_**	0.002037	0.001861	0.000764	0.002502	0.001398	0.000581
	**HRI**	0.006791	0.006203	0.002548	0.008341	0.00466	0.001936
Mn (R*f*D = 0.14)	**EDI_BW_**	0.008124	0.007378	0.006466	0.043143	0.017535	0.010253
	**HRI**	0.058028	0.0527	0.046187	0.308164	0.125247	0.073234
Cr (R*f*D = 0.003)	**EDI_BW_**	0.000265	0.000341	0.001459	0.00157	0.000083	0.000454
	**HRI**	0.088223	0.113652	0.486246	0.523397	0.027814	0.151333
Se (R*f*D = 0.005)	**EDI_BW_**	0.00008	0.000042	0.000072	0.000101	0.00003	0.000087
	**HRI**	0.016061	0.00835	0.014472	0.020196	0.006007	0.017484
Mo (R*f*D = 0.005)	**EDI_BW_**	0.000007	0.000008	0.000006	0.000019	0.000007	0.000012
	**HRI**	0.001313	0.001546	0.001279	0.003879	0.001364	0.002323
Co (R*f*D = 0.0003)	**EDI_BW_**	0.000015	0.000037	0.000067	0.000037	0.00001	0.00002
	**HRI**	0.049521	0.124754	0.223107	0.12494	0.031681	0.067146
Ni (R*f*D = 0.02)	**EDI_BW_**	0.000196	0.000192	0.00077	0.000795	0.00004	0.00023
	**HRI**	0.009811	0.009592	0.038497	0.039736	0.001977	0.011521
As (R*f*D = 0.0003)	**EDI_BW_**	0.000006	0.000003	0.000006	0.000011	0.000005	0.000024
	**HRI**	0.020747	0.009076	0.018335	0.035316	0.01538	0.07893
Cd (R*f*D = 0.001)	**EDI_BW_**	0.000001	0.000001	0.000002	0.000002	0.000001	0.000003
	**HRI**	0.001003	0.001342	0.001767	0.002385	0.001467	0.002715

R*f*D = oral reference dose (mg/kg/body weight/day) according to USEPA (2013).
